# Genome Sequence of *Virgibacillus pantothenticus* DSM 26^T^ (ATCC 14576), a Mesophilic and Halotolerant Bacterium Isolated from Soil

**DOI:** 10.1128/genomeA.01064-15

**Published:** 2015-09-17

**Authors:** Jie-ping Wang, Bo Liu, Guo-hong Liu, De-ju Chen, Yu-jing Zhu, Zheng Chen, Jian-mei Che

**Affiliations:** Agricultural Bio-Resources Research Institute, Fujian Academy of Agricultural Sciences, Fuzhou, Fujian, China

## Abstract

*Virgibacillus pantothenticus* DSM 26^T^ is a Gram-positive, spore-forming, aerobic, mesophilic, and halotolerant bacterium. Here, we report its 4.76-Mb draft genome sequence, which is the first genome information of *V. pantothenticus* and will promote biological research and biotechnological application for the species.

## GENOME ANNOUNCEMENT

As early as 1950, the type strain DSM 26^T^ (=B0018 = ATCC 14576 = CCUG 7424 = CFBP 4270 = CIP 51.24 = HAMBI 476 = JCM 20334 = LMG 7129 = NBRC 102447 = NCIMB 8775 [formerly NCDO 1765] = NCTC 8162 = NRRL NRS-1321 = VKM B-507) was isolated from soil and named *Bacillus pantothenticus* (n. sp.), because most strains require the intact pantothenic acid molecule for growth (1). In 1998, Heyndrickx et al. (2) established the new genus *Virgibacillus* to accommodate *B. pantothenticus* and reclassified *B. pantothenticus* as *Virgibacillus pantothenticus* comb. nov. Until now, there have been only two functional genes reported from *V. pantothenticus*: one is a type II restriction endonuclease, BpnI (3), and the other is a certain protease (4). Several researches indicated that ectoine and hydroxyectoine were protectants of *V. pantothenticus* against osmotic and cold stress (5–7). More recently, *V. pantothenticus* (*B. pantothenticus*) was reported to be a clinical opportunistic pathogen causing a liver abscess and sepsis (8, 9).

Given the taxonomic history, physiological and biochemical characteristics, and absence of genomic information available for *V. pantothenticus*, its type strain DSM 26^T^ was selected as one of the research objects in our “Genome sequencing project for genomic taxonomy and phylogenomics of *Bacillus*-like bacteria.” Here, we present the high-quality draft genome sequence of *V. pantothenticus* DSM 26^T^ (ATCC 14576).

The genome sequence of *V. pantothenticus* DSM 26^T^ was obtained by paired-end sequencing on the Illumina HiSeq 2500 system. Two different DNA libraries with insert sizes of 500 and 5,000 bp were constructed and sequenced. After filtering of the 1.31 Gb of raw data, the 1.20 Gb of clean sequence data were obtained, providing approximately 150-fold coverage. The reads were assembled via the SOAP*denovo* software version 2.04 (10). Through the data assembly, 16 scaffolds consisting of 4,759,248 bp were obtained, and the scaffold *N*_50_ was 2,767,993 bp. The average length of the scaffolds was 297,453 bp, and the longest and shortest scaffolds were 2,767,993 bp and 530 bp, respectively. Moreover, 89.81% clean reads were aligned back to the genome, by which 99.73% of the genome sequence was covered.

Annotation of the genome was performed using the NCBI Prokaryotic Genomes Automatic Annotation Pipeline (PGAAP) with the GeneMark, Glimmer, and tRNAscan-SE tools (11). A total of 4,274 genes were predicted, including 3,934 coding sequences (CDSs), 274 pseudogenes, 61 tRNAs, and 4 rRNA genes. There were 1,539 and 3,639 genes assigned to the COG and KEGG databases, respectively. The average DNA G+C content was 37.22%, in accordance with the previously acquired value of 36.9 mol% (*T*_m_) (1).

### Nucleotide sequence accession numbers.

This whole-genome shotgun project has been deposited at DDBJ/EMBL/GenBank under the accession no. LGTO00000000. The version described in this paper is version LGTO01000000.
